# Discharge from secondary care services to primary care for adults with serious mental illness: a scoping review

**DOI:** 10.1186/s12888-024-06067-6

**Published:** 2024-09-13

**Authors:** Aubrey L. W. Davis, Kennedy A. Hamilton, Jaclin A. Vozza

**Affiliations:** School of Rehabilitation Science, McMaster University, Hamilton, 1280 Main St. West, Hamilton, L8S 4L8 ON Canada

**Keywords:** General practitioners, Mental health, Mental illness, Psychiatry, Transition

## Abstract

**Background:**

Effective transitions of patients from Secondary Care Services (SCSs) to primary care are necessary for optimization of resources and care. Factors that enable or restrict smooth transitions of individuals with Serious Mental Illness (SMI) to primary care from SCSs have not been comprehensively synthesized.

**Methods:**

A scoping review was conducted to answer the questions (1) “What are the barriers and facilitators to discharge from SCSs to primary care for adults with SMI?” and (2) “What programs have been developed to support these transitions?”.

**Results:**

Barriers and facilitators of discharge included patient-, primary care capacity-, and transition Process/Support-related factors. Patient-related barriers and facilitators were most frequently reported. 11 discharge programs were reported across the evidence sources. The most frequently reported program components were the provision of additional mental health supports for the transition and development of care plans with relapse signatures and intervention plans.

**Conclusions:**

Established discharge programs should be comprehensively evaluated to determine their relative benefits. Furthermore, research should be expanded to evaluate barriers and facilitators to discharge and discharge programs in different national contexts and models of care.

**Trial Registration:**

The protocol for this scoping review is registered with the Open Science Framework (10.17605/OSF.IO/NBTMZ).

**Supplementary Information:**

The online version contains supplementary material available at 10.1186/s12888-024-06067-6.

## Background

 Secondary Care Services (SCSs) for adults with Serious Mental Illness (SMI) frequently use high-intensity care, such as Assertive Community Treatment, case management, and intensive case management models [1]. Although SCSs may sometimes refer to inpatient services, here we define SCSs as community and outpatient mental health services. (2, 3) Where appropriate, transitioning patients to lower levels of care allows for resources to be redirected to individuals whose care needs match those offered by SCSs; this then opens up access to appropriate levels of care across the broader care pathway [3–5]. For example, challenges accessing SCSs result in increased utilization and overburdening of tertiary care services which ultimately reduces access to crisis services, such as emergency services and crisis response teams [4, 7]. Inefficiencies within the healthcare system negatively impact patient care, care providers, and health economies [4]. SCSs supporting individuals with SMI note challenges with patient discharge to Primary Care Providers (PCPs) and broader issues with patient flow [6–10]. 

Despite the extensive impact of poor patient flow through SCSs, factors that enable or disable smooth transitions have not been comprehensively synthesized. Although the importance of transitioning individuals with SMI to lower levels of care has been discussed in the literature, there are no directive guidelines [7, 12]. A review conducted by the National Institute for Health and Care Excellence found no high-quality evidence with outcomes related to successful transitions to a lower level of support [13]. Blasi and colleagues [14] conducted a rapid review of the existent literature on discharge from SCSs to PCPs; however, limitations to this review are the limited number of databases searched and broad outpatient populations. Kim and colleagues [15] intended to conduct a scoping review of barriers and facilitators for transitions from specialty mental health services to primary care from the years 2000–2016. However, due to finding a small number of applicable studies, this scoping review was expanded to transitions from any specialty service to primary care [15]. Additionally, neither group of researchers focused specifically on adults with SMI [14, 15]. 

It should be noted that Assertive Community Treatment (ACT) and Early Psychosis Intervention (EPI) have been extensively researched in other papers [15–18]. Therefore, the purpose of this scoping review was to identify and map the extent of available research on discharge from other SCSs to PCPs for adults with SMI. Two specific research questions were addressed:

1. What are the barriers and facilitators for transitioning adults with SMI from SCSs to PCPs, as noted in scholarly literature since the year 2000?
2. What programs, services, or models have been developed to support transitions from SCSs to PCPs for adults with SMI, as noted in scholarly literature since the year 2000?

A scoping review was chosen as the method to address this subject because the authors were unable to find a review of the literature on this specific topic, and it was expected that there may be a mix of qualitative and quantitative data to consider as well as a variety of perspectives (e.g., patients, PCPs, SCS providers) [20]. Furthermore, a scoping review can assist in identifying gaps in the existing literature to clarify future research priorities related to improving the quality of transitions from secondary to primary care for this population [20]. 

Most definitions of SMI are operationalized through level of functional impairment, duration of impairment, or diagnosis, but usually include diagnoses where psychosis is a defining feature or a common symptom (i.e., schizophrenia, schizoaffective disorder, bipolar disorder, major depressive disorder with psychotic features) [21]. For practicality, this review will define SMI by the aforementioned diagnoses alone. Factors identified as influencing discharge to PCPs in one setting may not be applicable to different environments given the global diversity in institutional, political, economic, and cultural landscapes. Nevertheless, a comprehensive picture of the available research, and all potentially relevant factors can be obtained by considering available research internationally. The research questions specified literature from the year 2000 onwards because healthcare systems globally have undergone changes and older sources of information may no longer be applicable. Finally, this scoping review considered only peer-reviewed, scholarly sources due to time limitations. Quantitative, qualitative, and mixed-methods sources as well as peer-reviewed sources that did not report on a research study (e.g., a description of a program) were all considered for inclusion in this scoping review.

## Methods

The authors developed a scoping review protocol based on the Joanna Briggs Institute (JBI) guidance for scoping reviews [20]. The JBI guidance for scoping reviews was informed by previous work from Arksey and O’Malley [22] and Levac and colleagues [23] and aligns with the Preferred Reporting Items for Systematic reviews and Meta-Analyses extension for Scoping Reviews (PRISMA-ScR) [24]. The protocol for this scoping review is registered with the Open Science Framework (10.17605/OSF.IO/NBTMZ).

### Search strategy

Database searches were conducted in CINAHL, Embase, Emcare, MEDLINE, PsycINFO, and Web of Science from April 6 to April 17, 2023, to identify relevant peer-reviewed, published studies. The search was repeated again on March 24, 2024 to update the findings. The search strategy was initially drafted by the research team and finalized in consultation with a librarian at McMaster University Health Sciences Library. Searches were conducted using a combination of key terms, such as serious mental illness, discharge, outpatient mental health, and primary care and were limited to sources published between 2000 and 2023. When possible, in the individual databases, searches were limited to peer-reviewed sources, English language, and adult population. As an example, the full search strategy for PsycINFO is presented in Appendix A.

### Inclusion and exclusion criteria

Only studies in the English language were included, as this is the language spoken by both reviewers. To be included, studies also had to be peer-reviewed, include an adult population (18+) with diagnoses of SMI, and report on discharge from a secondary mental health or addiction setting to a primary care setting. Inclusion criteria were identified to ensure that studies were relevant to the specific review purpose through limiting identification of setting (e.g., SCS, primary care) and patient population-specific factors (e.g., age, diagnosis) that influence discharge. As such, studies based in forensic or inpatient mental health settings were excluded. Conference abstracts, clinical opinion pieces (e.g., letters to the editor), and non-peer-reviewed sources were excluded.

### Study selection

All identified studies were imported to Covidence review software where duplicates were automatically removed. Screening was independently completed by two reviewers in two phases. In stage one, titles and abstracts of studies were screened for relevance. In stage two, the full text of studies that appeared relevant were accessed, reviewed, and screened for eligibility against the inclusion and exclusion criteria. The specific list of reasons for exclusion that was used in stage two of screening included wrong patient population (e.g., a sample that did not include individuals with SMI), paediatric population, wrong setting (e.g., inpatient or forensic mental health), wrong outcomes, conference abstract, and wrong design (e.g., a letter to the editor). Studies that included both individuals with diagnoses of SMI and individuals without diagnoses of SMI were included since many of the secondary care settings in the identified studies served clients with a variety of diagnoses. Studies focusing on early intervention settings were included if it could be clearly identified that the participants of the studies were 18 years old or older at the time of discharge. Studies were excluded where study participant diagnoses were not identified or individuals using antipsychotic medications were excluded, as this suggested that individuals with SMI may not be included in the study. Any disagreements between the reviewers at both stages of screening were resolved through discussion and inclusion of the third researcher. Once all screening was completed, the reviewers searched the reference lists of included studies to identify additional studies that met inclusion and exclusion criteria and these were subsequently imported to Covidence. The reviewers also screened the reference lists of review papers that were relevant but did not meet the above eligibility criteria.

### Data charting

A data extraction template (see Appendix B) was developed by the researchers to extract data relevant to the scoping review questions from the included studies. The template was initially tested on five studies and revised to include the addition of columns titled “Facilitated Discharge Program,” and “Key Components of Facilitated Discharge Program.” Facilitated discharge programs/models/services reported in the evidence sources could be interpreted as facilitators of discharge. However, some sources described a program without conducting a research study or the research study was predicated on the use of a particular program, service, or model of care developed to facilitate discharge to primary care. Additionally, a number of studies that reported on facilitated discharge programs did not provide data to empirically support whether the program did in fact support discharge processes or outcomes. As a result, for this review programs were considered separate from the other facilitators reported in the literature. It was at this point that the second research question was added to this scoping review (“What programs or services have been developed to support transitions?”).

Data items charted for each study included: the authors, date, country of origin, the objective of the study, the study design (quantitative, qualitative, or mixed methods), the SCS setting from which patients were discharged (for example, a Community Mental Health Service), the sample population and/or participants of the study (patients, PCPs, etc.), barriers and facilitators to discharge to primary care, the name of the facilitated discharge program described, if applicable, and key components of the facilitated discharge program (information related to who/what/where/when factors). For the purpose of this scoping review, barriers were considered to be factors associated with reduced likelihood of discharge or unsuccessful discharge, and/or were quantitatively measured or stakeholder-perceived (qualitative) factors limiting the success of discharge to primary care (i.e., continued engagement with primary care after discharge from SCS). Facilitators were defined as factors associated with increased likelihood of discharge or successful discharge to primary care, and/or were quantitatively measured or stakeholder-perceived factors (qualitative) improving the success of discharge.

Two researchers independently completed data charting for each included study. Upon comparing the individually generated data charts, any disagreements were resolved through discussion. Critical appraisal of the sources of evidence was not completed, since the purpose of this scoping review was to identify the extent of research available rather than the quality of research [20]. 

### Data synthesis

Qualitative content analysis was used to synthesize the findings of this scoping review in relation to both research questions because this type of analysis can be applied to both quantitative and qualitative data and is appropriate for scoping reviews [20, 25]. This review used content analysis methodology outlined by Erlingsson and Brysiewicz [26], Vaismoradi and colleagues [27], and Kleinheksel and colleagues [25]. Two researchers independently reviewed the data from the data extraction tables and developed codes in an inductive and iterative process. Codes were then compared, refined, and organized into overarching categories and subcategories. When comparing codes, disagreements were first resolved through discussion and, where necessary, through involvement of a third reviewer. Frequency counts were also generated for codes from both research questions.

## Results

### Characteristics of included sources

**Fig. 1:**
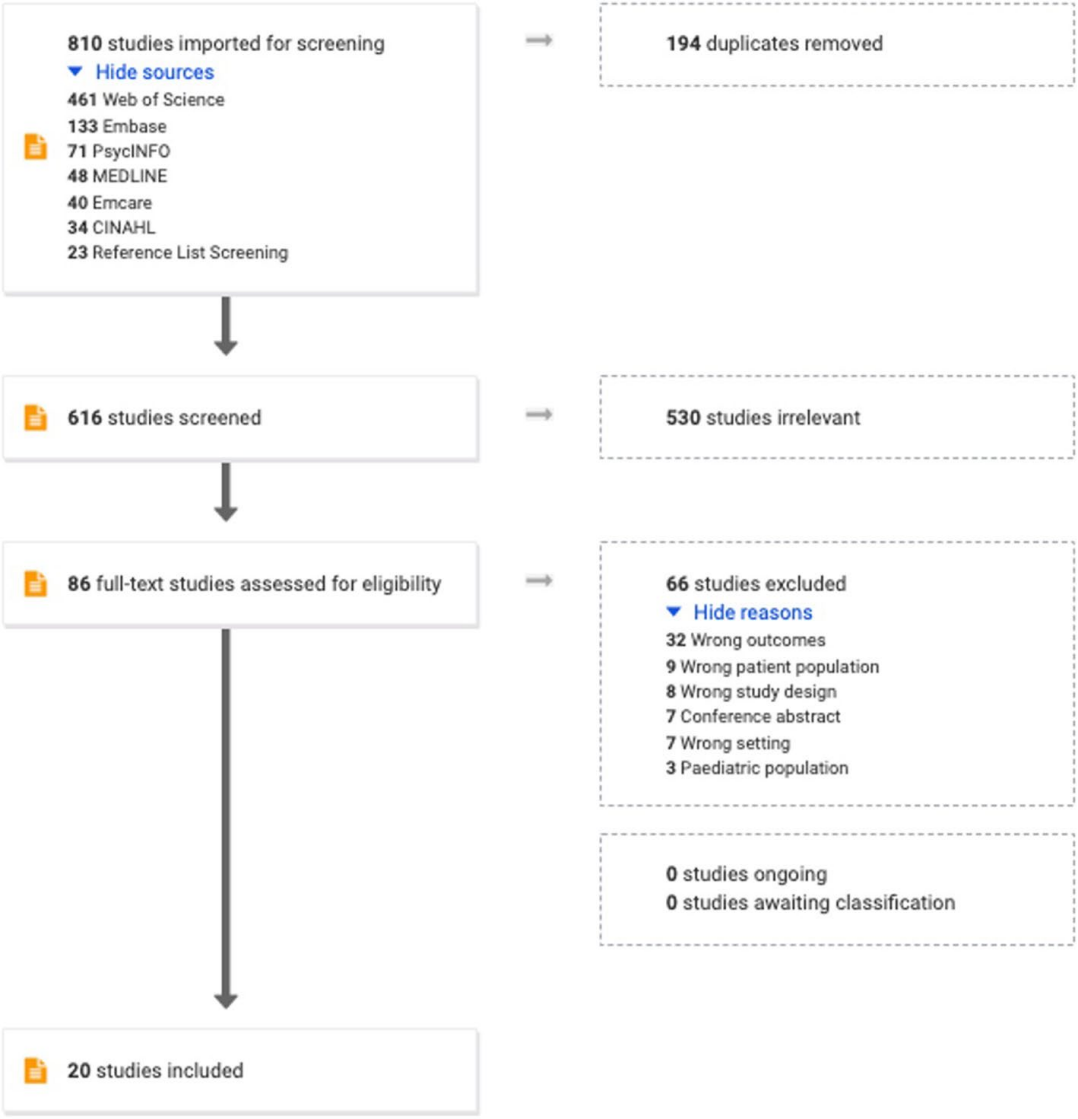
Prisma flow diagram. PRISMA flow diagram outlining the research and article screening process including reasons for study exclusion

 After duplicates were removed, the database searches yielded 593 unique sources of evidence. 530 studies were identified as irrelevant based on screening of their title and abstract, and the 63 remaining full texts were assessed for eligibility. Hand-searching of reference lists of all sources that met eligibility criteria and relevant review papers resulted in an additional 23 sources to screen. Thus, in total 86 full texts were assessed for eligibility. 66 sources were excluded at this stage. Exclusion reasons were outcomes unrelated to barriers and facilitators to discharge to primary care (*n* = 32), wrong patient population (*n* = 9), wrong study design (*n* = 8), wrong setting (*n* = 7), conference abstract (*n* = 7), and paediatric population (*n* = 3). Data charting was completed for the remaining 20 sources that met the eligibility criteria. See Fig. 1 for the Preferred Reporting Items for Systematic reviews and Meta-Analyses (PRISMA) flow diagram.

Of the 20 included sources, the majority were quantitative research studies (*n* = 11) or mixed methods studies (*n* = 6). Of the quantitative studies (*n* = 11), 2 were cross-sectional surveys, 6 were retrospective chart reviews, audits, or cohort studies, 2 were program description and outcome evaluations, and 1 was a prospective service evaluation. Additionally, two sources were qualitative studies and one source described a program without reporting on research findings. Of the qualitative studies, 1 involved semi-structured interviews with care providers, while the other involved semi-structured interviews with individuals with psychosis. For sources reporting on research (*n* = 19), most studies included participants from multiple stakeholder groups (for example, patients, PCPs, clinicians from SCSs, and others; *n* = 8). Many studies used patients’ charts as data sources (*n* = 8) and others collected data only from patients directly (*n* = 3). The countries of origin of the research studies and programs included in this review were Australia (*n* = 7), the United Kingdom (*n* = 6), New Zealand (*n* = 3), the Netherlands (*n* = 2), Ireland (*n* = 1), and Canada (*n* = 1). Due to the heterogeneity in healthcare systems in different countries and differences in the clarity of reporting settings across the sources, it was difficult to group and quantify the SCSs reported. SCSs included Community Mental Health Centres, Community Mental Health Teams, Community Mental Health Services, Public Mental Health Services, specialist mental health services, public psychiatric services, early intervention for psychosis services, and other settings not well defined. Although addiction settings were considered in this scoping review, no sources focused on addiction settings met all the eligibility criteria for inclusion.

### Barriers and facilitators to discharge

Study extraction data were coded within the broader categories of barriers and facilitators to discharge. Individual codes were then subcategorized into patient-related, primary care capacity-related, and transition Process/Support-related barriers or facilitators to discharge (see Table 1 for full coding for this research question). See Appendix D for a summary coding table for this research question. Some data associated with discharge to primary care from the included evidence sources was not categorized as barriers or facilitators to discharge because of contradictory information across sources. For example, Filia and colleagues [28] reported that those with longer illness duration and longer time taking clozapine were more likely to be discharged to primary care than to private psychiatry. However, Jespersen and colleagues [29] reported that less chronicity was associated with discharge, and Ramanuj and colleagues [30] reported less time spent with secondary care was associated with discharge. Furthermore, Filia et al. [28] and Jespersen et al. [29] both found that those with fewer contacts with SCSs were more likely to be discharged. Thus, it was decided that within the context of this scoping review, which aims to map evidence rather than interpret it, duration of SMI and time spent within a SCS could not be categorized as barriers or facilitators. Additional miscellaneous factors associated with discharge (female sex, diagnoses of less high prevalence disorders, and fewer family contacts [29]) were also not categorized.

**Table 1:** Barriers and facilitators of discharge

Barriers		
Patient Related	Primary Care Capacity Related	**Transition Process/Support** Related
**Patient stability** - Recent symptom onset - Beckers 2019 - Recent acute crisis care - Ramanuj - History of high risk events - Castelino 2016 **Care needs** - High need for secondary care services - Filia 2012, Stangroom 2014 - High risk symptoms - Beckers 2019 - High symptom load - Filia 2013 - Psychosocial impairment - Filia 2013 - Higher substance use - Filia 2013 - Need for medications - Ramanuj 2015 **Socioeconomic status** - Limited support network - Beckers 2019 - Homelessness - Holmes - Ability to pay for PCP services in private or semi-private models of care - Agyapong 2012 **Engagement with treatment** - Low motivation to engage in treatment - Beckers 2019 - CTO - Filia 2013 - Medication and/or treatment compliance - Filia 2012, Filia 2013, Stangroom 2014 **Readiness for discharge** - Unexpected or abrupt discharges - Lester 2012 - Feeling passed on/over by early intervention services - Lester 2012 - Concern about loss of contact with a psychiatrist - Lester 2012, Rodenburg 2004	**Accessibility and care context-related factors** - Patient low levels of personal organizational skills impacting self-management - Filia 2012 - Less patient accountability for attending appointments in primary care - Filia 2012 - Stress related to unfamiliar environment of PCP - Rodenburg 2004 - Patient and PCP concerns about time constraints in PC - Rodenburg 2004, Stangroom 2014 - Patient preference to remain in secondary care - Agyapong 2012 **PCP ability to meet patient needs** - Medication complexity - Beckers 2019, Filia 2012 - Need to establish therapeutic relationship - Filia 2012 - Patient concerns about quality of psychiatric care from PCPs - Agyapong 2012, Rodenburg 2004	**Quality of communication and support across care settings** - Poor communication across care settings - Stangroom 2014, Lester 2012 - Lack of information about transition process - Filia 2012 - Lack of support from PC/private psychiatry sector - Filia 2012 - Lack of support from secondary care services - Filia 2012, Stangroom 2014 **Work and time required to facilitate discharge** - Time required for transition process - Filia 2012 - Amount of paperwork required - Filia 2012
Facilitators		
Patient Related	Primary Care Capacity Related	**Transition Process/Support** Related
**Stability** - General stability - Castelino 2016, Beckers 2019 - Functional remission of SMI/high overall functioning - Beckers 2018, Beckers 2019, Jespersen 2009, Ramanuj - Employment - Jespersen 2009 - Fewer medical conditions - Jespersen 2009 - Less medication needs - Ramanuj, Jespersen 2009 - Less acute care time/encounters - Ramanuj, Jespersen 2009 - Absence of substance abuse - Filia 2012 - Less psychosocial stress - Jespersen 2009 - Less current/past CTO - Jespersen 2009 **Patient strengths** - Strong support systems - Beckers 2019, Beckers 2018 - High motivation - Beckers 2019, Beckers 2018 - Medication compliance - Filia 2012 - Presence of skills - Beckers 2018, Beckers 2018 - Good cognitive function - Castelino 2016 - Insight into illness - Beckers 2018, Castelino 2016 - Ability to attend appointments and blood tests independently - Filia 2012 **Readiness for discharge** - Feeling prepared for discharge - Lester 2012 - Patient aware of and expecting discharge - Lester 2012, Rodenburg 2004 - Patient approval of discharge - Beckers 2018, Filia 2012 - Patient faith in transfer of care - Beckers 2018 - Patient not feeling pressured to transfer - Rodenburg 2004 - Patient viewing PC as less stigmatizing - Agyapong 2012	**Accessibility and care context-related factors** - Accessibility and convenience of PC - Agyapong, 2012, Rodenburg, 2004, Filia 2012 - Physical access to a pharmacy - Filia 2012 - Ability to access services for free or to afford services in semi-private or private healthcare systems - Agyapong 2012, Rodenburg, 2004, Filia 2012 - Patient preference for primary care - Agyapong, 2012 **PCP ability to meet mental health care needs** - Patient belief in PCP ability to meet needs - Agyapong 2012 - Previous strong and trusting relationship between patient and PCP - Lester 2012 - PCP ability to recognize need for additional services - Lester 2012 - Welcoming environment in PCP office - Rodenburg 2004 - Ability for patient to receive coaching and mental health monitoring - Baker 2019 - Ability to have consistent contact with patient - Baker 2019	**Discharge planning process** - Planned process involving interdisciplinary team - Backus, Horner & Asher, 2005 - Inclusion of PCP in discharge planning process - Horner & Asher, 2005 - Inclusion of patient in discharge planning and care plan development - Rodenburg, 2004, Lester, 2012 - Personalized and flexible discharge process - Lester, 2012 - Transparency in discharge planning process - Rodenburg 2004 - Collaborative development of care plan - Horner & Asher, 2005 - Recognition of patient’s self-management ability - Lester, 2012 **Communication and support across services** - Ongoing communication and support between primary, secondary care, and patient - Baker 2019, Horner & Asher, 2005, Stangroom 2014, Lester 2012, Hamilton-West 2017 - Organization of services - Becker 2018 - Facilitated re-entry or access to secondary care services when needed - Filia 2012, Lester 2012 - Primary and secondary care healthcare providers having faith in the transfer (Beckers et al., 2018)

### Patient-related barriers

Regarding barriers, patient-related codes included patient stability, care needs, socioeconomic status, engagement with treatment, and readiness for discharge. Patient stability was noted in three sources as recent onset of symptoms [31], a history of high-risk events [32], and recent use of acute crisis care [30]. Care needs were reported in five sources and included a high need for SCSs [33, 34], high-risk symptoms [31], high symptom load [28], psychosocial impairment [28], high substance use [28], and need for medications [30]. Socioeconomic status factors, reported in three sources, included having a limited support network [31], experiencing homelessness [35], and difficulties paying for primary care services in private or semi-private models of care [36]. Issues related to engagement with treatment posing barriers to discharge were reported in four sources as low motivation to engage in treatment [31], low medication and/or treatment compliance [28, 33, 34], and having a Community Treatment Order (CTO; [28]). Finally, factors related to readiness for discharge were reported in two sources as having an unexpected or abrupt discharge [37], “feeling passed on” by the SCS, or being discharged without feeling ready or without having the appropriate supports in place [37], and concerns about losing contact with a psychiatrist [11, 37].

### Primary care capacity barriers

Primary care-related codes for barriers were accessibility and care context-related factors and PCP ability to meet patient needs. Accessibility and care context-related factors were reported in four sources and included factors such as clinicians perceiving patients to have a low level of personal organizational skills impacting self-management [33], primary care having less patient accountability [33], patient fears about unfamiliarity and stressors in the physical environment of primary care [11], patient and PCP concerns about time constraints in primary care [11, 34], and patient preference to remain in secondary care [36]. Factors related to PCP ability to meet patient needs were reported in four sources and included the factors of patient concerns about quality of psychiatric care from PCPs [11, 36], managing medication complexity [31, 33], and the need to establish a therapeutic relationship [33].

### Transition process/support-related barriers

Process/systems-related codes included quality of communication and support across care settings and work and time required to facilitate discharge. Communication and support factors, reported in three studies, included poor communication between secondary and primary care [34, 37], lack of support from and/or between PCPs and SCSs [33, 34], and a lack of information about the transition process [33]. The work and time required to facilitate discharge was noted in one source [33] as time required for the transition process and the amount of paperwork required.

### Patient-related facilitators

Codes for patient-related facilitators included stability, strengths, and readiness for discharge. Stability factors as facilitators were reported in six sources as general stability [31, 32], functional remission of SMI or high overall functioning [28–30, 38], having employment [29], having fewer medical conditions [29], having fewer medication needs [30], having less psychosocial stress [29], having an absence of substance abuse [33], having less time spent in or fewer encounters with acute care [29, 30], and not having or had a CTO [29]. Patient strengths were reported in four sources as having a strong support system [31, 38], high motivation [31, 38], skills [31, 38], medication compliance [33], good cognitive function [32], insight into their SMI [32, 38], and the ability to attend appointments and blood tests independently [33]. Readiness for discharge factors, reported in five sources, included feeling prepared for discharge [37], being aware of and expecting discharge [11, 37], approving of the discharge [33, 38], having faith in the transfer of care [38], viewing primary care as less stigmatizing [36], and not feeling pressured to be discharged [11].

### Primary care capacity facilitators

Primary care-related codes for facilitators were accessibility and care context related-factors and PCP ability to meet mental health care needs. Accessibility and care context related-factors, reported in three sources, included patient preference for primary care over SCSs [36], the accessibility and convenience of primary care for patients [11, 33, 36], patients’ physical access to a pharmacy [33], and patient ability to financially access primary care services in semi-private or private healthcare systems [36]. Factors related to PCP ability to meet mental healthcare needs were reported in four sources as patients’ belief in PCPs’ ability to meet their needs [36], PCPs’ ability to recognize when additional support is needed [37], the ability for patients to receive coaching, mental health monitoring, and consistent contact within primary care [39], having an established strong and trusting relationship between the patient and PCP [37], and the primary care office having a welcoming environment [11].

### Transition process/support-related facilitators

Codes for process/systems-related facilitators were the discharge planning process and communication and support across services. Four sources reported factors related to the discharge planning process, including having a collaborative and planned process involving an interdisciplinary team [8, 9], including PCPs in the discharge planning process [9], including patients in discharge planning and care plan development [11, 37], having a personalized and flexible discharge process [37], having transparency in the discharge process [11], and recognizing patients’ self-management ability when planning discharge [37]. Seven sources reported factors related to communication and support across services including having ongoing communication and support between primary care, SCSs, and patients [7, 9, 34, 37, 39], facilitated re-entry or access to SCSs when needed [33, 37], and primary care and SCS healthcare providers having faith in transfers of care [38].

### Facilitated discharge programs

11 facilitated discharge programs were reported across 12 of the evidence sources: the Transition into Primary Care Psychiatry (TIPP) clinical model [40], the Consultation and Liaison in Primary Care Psychiatry (CLIPP) model [29, 41], the Recovery and Enablement Track [10], the Enhanced Primary Care (EPC) Pathway [42], a primary care liaison service [29], the Wellington Mental Health Liaison Service [11], a modified shared care protocol [9], the Primary Care Mental Health Specialist (PCMHS) Service [7], a shared care model for clozapine [28, 33], the PARTNERS (develoPing integrAted primaRy care for paTieNts with sERiouS mental illness) program [39], and a planned discharge process [8]. Of the programs, five were developed and employed in Australia [8, 9, 28, 29, 33, 41], four in the United Kingdom [7, 10, 39, 42], one in Canada [40], and one in New Zealand [11]. Funding for these individual programs depends on the model of care and country of origin.

**Fig. 2:**
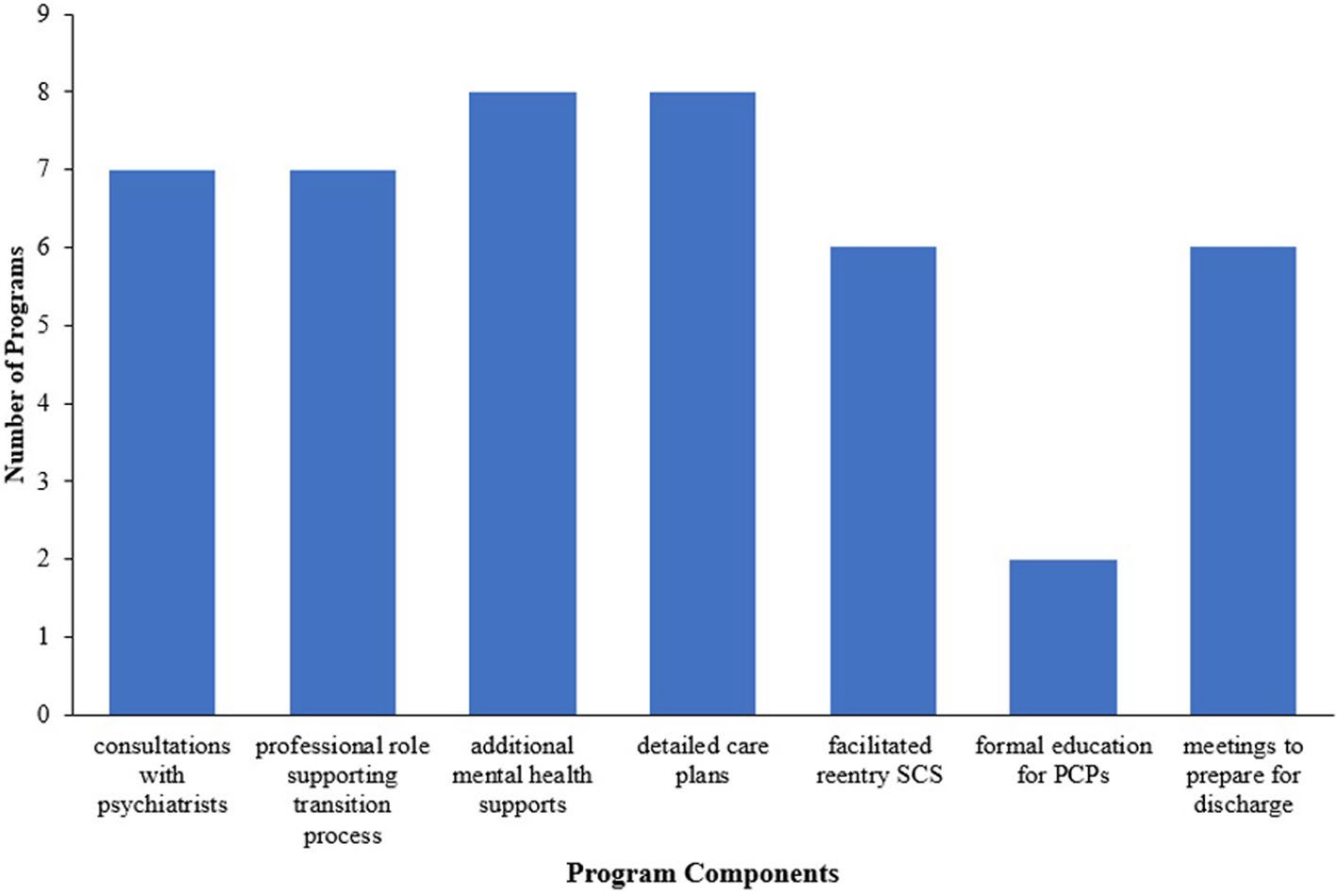
Common components of identified discharge programs. Bar graph illustrating the frequency of common program components within the eleven programs that aim to facilitate discharge of individuals with SMI from SCSs to primary care

 For this research question, the major components of discharge programs were ascribed codes (see Appendix E for coding table). Figure 2 demonstrates the frequency of common components among the 11 discharge programs.

A new professional role supporting SCSs, PCPs, and patients through the transition of patient care from SCSs to PCPs was identified in seven programs across nine evidence sources [8, 9, 28, 29, 33, 38–41]. The development and provision of a detailed care plan, including relapse signatures and relapse interventions plans, was integrated into eight programs [6–8, 11, 29, 38–41]. Likewise, provision of mental health supports distinct from PCPs was identified as a component in eight programs [7, 8, 10, 28, 29, 33, 39, 40, 42]; these supports involved supports like case management, recovery-oriented groups, peer support, psychoeducation, and self-management. Consultations with psychiatrists were available within seven programs, meaning that PCPs could consult with psychiatry about clinical questions; this was either structured or on an as-needed basis depending on the agency [9, 28, 29, 33, 38–41]. Facilitated re-entry into a SCS was a component of six programs [7, 9, 10, 28, 29, 33, 42]. Six programs also included discharge planning meetings with various relevant stakeholders [8, 9, 11, 29, 40, 41]. Lastly, two programs provided formal education/training programs to PCPs [11, 42]. Primarily, transition programs were delivered across the boundaries of secondary and primary care services; however, one program was delivered in the context of a SCS, focusing on patient readiness for transfer of care [10], and another was delivered in the context of a SCS to improve processes related to patient transfer [8].

## Discussion

This scoping review identified various barriers and facilitators to discharge from SCSs to primary care (patient-, primary care-, and process/systems-related factors) as well as 11 facilitated discharge programs. The most frequently noted facilitators and barriers to discharge from SCS to PCP within the reviewed literature were patient-related factors. In total, primary care-related barriers and facilitators and systems/process-related barriers and facilitators were identified an equal number of times across publications. While the most common category of facilitators and barriers was patient-related factors, the single most frequently noted factor across patient-, primary care-, and process/systems-related categories was communication and support across services as a facilitator to discharge. Although being the most commonly identified factor across research studies does not necessarily indicate relative importance, the frequency with which factors were identified suggests relative consensus of their importance. Consensus on the importance of patient stability and communication across services is exemplified by additional mental health supports to the patient while being followed by primary care, and detailed care plans being the most frequently reported elements of discharge programs.

The programs identified within this review plausibly address many of the modifiable barriers to discharge identified within this review. Consultations with psychiatrists may allow for increased PCP knowledge of management of patient’s SMI, comfort with medication, and contribute to maintenance of patient stability while in PCP care. Professional roles supporting the transition process may facilitate increased communication between PCPs, SCS, and patients, enhance PCP knowledge of management of patient’s SMI, maintain or enhance patient’s readiness for the transition, and help bolster patients’ engagement with treatment. Additional mental health supports may improve patient stability, aid in the development of patient strengths, maintain or increase readiness for discharge, and contribute to reduced direct care required from PCP. Detailed care plans may also facilitate communication between PCPs, SCSs, and patients, improve PCP knowledge of management of patient’s SMI, and promote increased faith in the discharge. Likewise, facilitated re-entry into SCSs acts as a support to discharged patients and PCPs and may increase faith in the discharge. Formal education for PCPs may improve PCP ability to meet patient care needs, improve PCP confidence in SMI treatment, and improve faith in the discharge. Finally, meetings with stakeholders in preparation for discharge allow for increased communication between PCPs, SCSs, and patients, and are suggested to allow for a smoother discharge planning process.

Although the discharge programs presumptively facilitate discharge, the overall effectiveness of identified programs is largely unreported in sources included in this review. The outcomes reported for the discharge programs are largely outside the scope of this review, but it is notable that no studies of the identified programs compared outcomes using a similar control group. Thus, conclusions cannot be made about the effectiveness of programs at facilitating successful discharges to primary care relative to standard practices. Where ethically tenable, future studies should evaluate outcomes of discharge programs using control groups to determine effectiveness on key indicators including function, health status, and subsequent service utilization. The costs of program implementation were evaluated for two of the identified programs [7, 11]; however, only one study identified how this compared to continued care within SCSs [11]. Furthermore, economic evaluations did not include costs associated with re-entry to SCSs or other subsequent transitions in care related to potential deterioration in health status and unmet care needs [11]. Given the potential costs of many of the identified programs and costs associated with transfer of care, outcome evaluations should be paired with comprehensive economic analysis to determine the relative benefits of different programs and their respective elements.

As noted by Filia and colleagues, a lack of information about the transition process is a barrier [33]. Although not included in this review due to its focus exclusively on the primary care context, a study by Fleury and colleagues [43] noted that difficulty facilitating patient access to specialized services and supports results in PCPs experiencing a sense of hopelessness. Where PCPs have minimal understanding of safeguards embedded in patient discharge processes, fears surrounding inability to provide adequate level of care and the potential for patient relapse may cause PCP reluctance to accept sole care. One study of PCP knowledge and use of community services in Toronto, Ontario demonstrated significant gaps in awareness of community services for individuals with mental illness and centralized intake services [44]. PCPs may also be unaware of current discharge processes and practices within SCSs for individuals with SMI. Further research should be conducted to determine the level of PCP knowledge of SCS discharge practices and processes and whether providing information on established safeguards improves PCP confidence in providing care to individuals with SMI. Greater outreach to PCPs to improve understanding of SCS discharge practices could be facilitated if significant knowledge gaps are identified as a barrier to PCP involvement/acceptance in patient discharge.

The preponderance of studies emanating from Australia and the United Kingdom raises the question of whether the same barriers and facilitators to discharge exist within the Canadian context and other national healthcare systems. Culture and/or healthcare system-related factors may influence facilitators and barriers to discharge. Furthermore, the utility of discharge programs may differ depending on contextual factors. In Ontario, Canada, several different models of primary care have been developed that may have unique barriers to discharge. For example, Family Health Teams use an interdisciplinary approach to primary care (which can include consultations with psychiatry), and while doctors may be remunerated through blended capitation models, other health care providers are salaried [45, 46]. Within Quebec, Canada, PCPs working at Health and Social Services Centres are salaried employees and work with an interdisciplinary team including mental health professionals [43]. Within these contexts, PCPs suggested that they were better able to follow-up with patients with SMI due to greater flexibility with time allocation relative to a fee-for-service model, and they were better equipped to care for individuals with SMI due to the presence of interdisciplinary supports [43]. Thus, the value of discharge programs may be dependent on the specific model of primary care. Future studies should investigate discharge outcomes and perceived barriers to discharge across different models of primary care and within the Canadian context.

### Implications and recommendations

Identifying and targeting modifiable factors affecting discharge to PCPs may assist SCSs, PCPs, and the broader healthcare system to improve transition processes. Effective communication between SCSs, PCPs, and patients and a coordinated discharge planning process could be implemented through various institutional initiatives to facilitate smooth transitions between primary and secondary care. SCSs could institute a policy that key stakeholders, including patients and PCPs, should be involved in a pre-discharge meeting to develop a care plan and address any foreseeable challenges. PCPs could be provided contact information to consult with specialized services on an as needed basis. Given the various models of primary care and their potential differences in barriers and facilitators, different initiatives may be of greater utility in different contexts, and there may be benefit to tailoring discharge practices to specific models of care. Although outside of the purview of the healthcare system, broader governmental policies addressing social determinants of health like housing first initiatives may also have an impact on the feasibility of discharge to PCP. Knowledge translation of the types of programs identified within the literature and their theoretical underpinnings should also be conveyed to SCSs and PCPs. For research, additional investigations should be conducted across different models of primary care, and international contexts. Furthermore, the effectiveness of discharge programs should be evaluated.

#### Strengths and limitations

Strengths of this scoping review included the consultation of a health sciences librarian for developing the search strategy, searching multiple databases, and screening reference lists of review papers for additional evidence sources to include in the review. The comprehensiveness of the search was limited, however, by including only peer-reviewed, English language sources. It is possible that there are perspectives from stakeholders captured in non-scholarly sources and other languages that would add information on the barriers and facilitators to discharge and programs used to support discharge. Furthermore, content analysis is a subjective process. Although two reviewers reached consensus on the coding and categorization of the codes, all factors could be interpreted in multiple ways

## Conclusion

Results of this scoping review indicate that whether discharge of patients with SMI from SCSs to PCPs is feasible may depend on several factors related to the patient, their PCP, and processes/systems. Many of the barriers identified within this review could plausibly be modified through implementation of targeted programs and practices. Although several programs have been developed that appear to address modifiable barriers to discharge, their effectiveness has not been established. Thus, established discharge programs should be comprehensively evaluated to determine their relative benefits. Furthermore, research should be expanded to evaluate barriers and facilitators to discharge and discharge programs in different national contexts and models of care.

## Supplementary Information

Supplementary Material 1.

## Data Availability

All data generated or analysed during this study are included in this published article.

## Abbreviations

SCS: Secondary Care Service
SMI: Serious Mental Illness
PCP: Primary Care Provider
PRISMA-ScR: Preferred Reporting Items for Systematic reviews and Meta-Analyses extension for Scoping Reviews
TIPP: Transition into Primary Care Psychiatry
CLIPP: Consultation and Liaison in Primary Care Psychiatry
EPC: Enhanced Primary Care
PCMHS: Primary Care Mental Health Specialist
PARTNERS: develoPing integrAted primaRy care for paTieNts with sERiouS mental illness
