# Supplementary material for: Discharge from secondary care services to primary care for adults with serious mental illness: a scoping review
Source: BMC Psychiatry. 2024 Sep 13;24:614. doi: 10.1186/s12888-024-06067-6 (PMC11396452; doi:10.1186/s12888-024-06067-6)
Supplement: Supplementary file 1 — Supplementary Material 1. [file 12888_2024_6067_MOESM1_ESM.docx]

### **Appendix A**

### Search Strategy for PsycINFO

Database: APA PsycInfo <1987 to April Week 2 2023>

Search Strategy:

--------------------------------------------------------------------------------

1 (((severe or serious) adj3 mental illness*) or Schizophrenia or schizoaffective or psychos?s or bipolar or psychotic or delusion* or psychiatric).mp. [mp=title, abstract, heading word, table of contents, key concepts, original title, tests & measures, mesh word] (379825)

2 (discharg* or transition* or transfer* or graduat* or mov*).mp. [mp=title, abstract, heading word, table of contents, key concepts, original title, tests & measures, mesh word] (460168)

3 (Outpatient mental health or addiction service* or outpatient psychiatry or community mental health or assertive community treatment or ACT or FACT or flexible assertive community treatment or early intervention or specialist or case management or secondary care or speciality mental health).mp. [mp=title, abstract, heading word, table of contents, key concepts, original title, tests & measures, mesh word] (208787)

4 (Primary care or primary healthcare or family doctor or general practitioner or GP or PCP or family health team).mp. [mp=title, abstract, heading word, table of contents, key concepts, original title, tests & measures, mesh word] (44305)

5 1 and 2 and 3 and 4 (150)

6 limit 5 to (peer reviewed journal and english language and adulthood <18+ years> and yr="2000 -Current") (71)

***************************

### **Appendix B**

| **Author(s), (year), country** | **Objective/Aim** | **Study**  **Design** | **Secondary Care Service Setting** | **Participants/Sample population** | **Barriers to**  **transition to primary care** | **Facilitators to transition to primary care** | **Facilitated Discharge Program** | **Key Components of Facilitated Discharge Program** |
| --- | --- | --- | --- | --- | --- | --- | --- | --- |
|  |  |  |  |  |  |  |  |  |
|  |  |  |  |  |  |  |  |  |

Appendix B. Data Extraction Template.

**Appendix C**

| **Author(s), (year), country** | **Objective/Aim** | **Study**  **Design** | **Secondary Care Service Setting** | **Participants/Sample population** | **Barriers to**  **transition to primary care** | **Facilitators to transition to primary care** | **Facilitated Discharge Program** | **Key Components of Facilitated Discharge Program** |
| --- | --- | --- | --- | --- | --- | --- | --- | --- |
| Agyapong (2012), Ireland | To explore patient preferences for primary care vs. specialized psychiatric services to attend to continuing mental health needs after achieving stability in psychiatric services | Quantitative: Cross-sectional survey: semi structured questionnaire completed by psychiatric patients investigating preferences regarding continuing mental health care once stable. Timing of questionnaire was pre-discharge | Community Mental Health Centre in Dublin | Patients (n = 145, 52% male, 15% - bipolar affective disorder diagnosis, 11% - schizophrenia or schizoaffective disorder diagnosis, M =  45.48 years old (SD = 13.01 years)). | Majority (68%) of individuals preferred continuing with specialized psychiatric services  Reasons provided were: they would be worried about the quality of the  psychiatric care from PCPs (68%); they do not have a medical/PCP visit card and cannot  pay for PCP consultation (41%); they do not have a PCP (9%) and other reasons (19%) | 20% indicated a preference for PCP care.  Reasons given were: they are confident their PCP can provide the same level of care as CMHC (62%); convenience in accessing physical and mental care in the same place (45%); it would reduce stigma attached to receiving psychiatric care (31%); use of medical card to obtain PCP services free of charge 5 (17%); and Other reasons (14%) |  |  |
| Backus et al. (2008), Australia | To explore the rate of adults needing acute mental health interventions after planned  discharge process from community mental health case management. | Quantitative: Retrospective chart review/audit: demographic factors and clinical variables were collected on all discharged patients at one year and then at three years following discharge | Wyndham Continuing Care Team (WCCT - case management) of Werribee Mercy Mental Health Programme (WMMHP) in Melbourne | Patients (n = 46, 73% female, 39 discharged to PCP, 47.8% - psychotic disorder, M = 42 years of age (range 23-66)) | No significant relationships found between demographics/clinical characteristics and use of acute mental health interventions after discharge | Planned discharge involving a multidisciplinary team increased the number of patients that could be discharged from services, from 11 in 2000 to 46 in 2001. |  |  |
| Baker et al., (2019), United Kingdom | To describe and evaluate the implementation and theoretical underpinnings of a collaborative care model for patients with psychotic disorders. | Qualitative: semi-structured interviews and review of recorded consultations with primary and secondary care providers discussing the PARTNERS model of collaborative care | Secondary care services operated by NHS trusts for three sites. | Patients (n = 16; 44% with schizophrenia diagnosis, 56% with bipolar affective disorder diagnosis; M(SD) = 53.3 (11.04) years of age; 75% male)  Family carers (n = 5)  Care partners (n = 3)  Supervisors (n = 3)  PCPs (n = 4)  Other primary or secondary health care workers (n = 6) |  | Stability of mental health within primary care was perceived to be improved through care partner liaison with secondary care services, coaching, consistency of contact with clients, and mental health monitoring. | PARTNERS (develoPing integrAted primaRy  care for paTieNts with sERiouS mental illness) | Psychiatrist consultation available if needed  Care Partner:  liaises between levels of care and provides direct support to clients  Supervisor: conducts meetings with care partners bimonthly to consult on care.  20-90 minute intervention sessions involving coaching, goal setting, and motivational interviewing and psychoeducation.  Care plan with relapse signature intervention  Formal training provided to care partner and supervisor  8-10 months  Shared records |
| Beckers et al. (2019), Netherlands | To explore the outcome of stable patients transferred from specialized mental health care services to primary care. | Mixed Methods: patients receiving specialized mental healthcare and involved professionals were engaged in a focus group just prior to the patients’ discharge. | Community Mental Health Teams | PCPs (n = 3)  Community Mental Health Nurses (n = 3)  Psychiatrist (n = 1)  Patients (n = 32, 63% male, 28% schizophrenia or other psychotic disorder diagnosis,  M = 47 years old (±10.8)) | All groups identified:   - Use of medications requiring complex follow-up (clozapine or lithium) - Low motivation - First years following first psychotic episode - Suicidal/ aggressive tendencies - Limited/precarious support network. | All groups identified:   - Functional and symptomatic remission - High motivation - Strong support systems   PCPs and patients emphasized:   - Patients’ skills - Support from family and friends   Psychiatrist and community mental health nurse emphasized:   - Professional support |  |  |
| Beckers et al. (2018), Netherlands | To identify factors that can guide determination of whether discharge from specialist mental services to lower levels of care will be successful. | Mixed Methods: Concept mapping – healthcare providers working with those who have SMI, GPs, peer workers, community mental health nurses, primary care nurses, and community social workers were told to generate statements about predictive factors for successful referral from specialized mental health services | Specialist Mental Health Services | General practitioners (n = 7)  peer workers (n = 8),  community mental-health nurses (n = 9)  neighborhood-based social workers (n = 11),  other healthcare professionals (n = 3) |  | From most to least predictive of success the clusters identified were: Patient related factors (such as motivation, awareness of their illness, and skills), the informal support system, the patient’s social situation, the organization of services, and the healthcare service providers faith in the transfer.  The most predictive individual statements, ordered from most to least, were: Whether the patient has faith in the transfer, The patient’s motivation, The patient’s approval and The phase of recovery the patient is in |  |  |
| Castelino et al. (2016), New Zealand | To identify factors psychiatrists use in determining whether patients  on depot antipsychotics (LAI) are suitable for discharge from CMHS to primary care. | Quantitative: Retrospective observational study of case file reviews; a questionnaire was developed to capture sociodemographic information, clinical characteristics of the patients, and clinical opinion on suitability of discharge to primary care. | Specialist adult community  mental health team of a District Health Board | Patients (n =72, 71% male, M age 40.67 (SD 13.68), 11 had a  schizophrenia diagnosis, 2 had a bipolar disorder diagnosis and 1 unipolar  Depression diagnosis) | History of high-risk events significantly associated with not being suitable for discharge | Stable mental state > 1 year and good  cognitive function and  insight significantly associated with suitability for discharge |  |  |
| Filia et al. (2013), Australia | To assess the effectiveness of discharge to PCP shared care, discharge to private psychiatric care, and continued CMHS support for managing  patients treated with clozapine. | Quantitative: Retrospective file audit – medical records for clozapine patients were audited for a 24-month period | Alfred Public Mental Health Service (PMHS; Melbourne) | Patients (n =90, individuals with schizophrenia or schizoaffective disorder taking clozapine) | Patients not discharged from the PMHS had higher substance use, were less compliant with medications, were more likely to be on a community treatment order, and displayed significantly more symptoms and psychosocial impairment than the discharged groups | Patients transitioned to PCP shared care had a significantly longer duration of illness than patients transitioned to private psychiatrists or continuing in PMHS  Patients transitioned to PCP shared care had been taking clozapine for longer than the other groups  Patients transitioned to PCP shared care or private psychiatry had lower numbers of contacts with PMHS case managers and psychiatrists compared to patients remaining in the PMHS | Shared Care Model for Clozapine | Psychiatrist completes a review every 6 months or when required  Clozapine coordinator: supports client and PCP and facilitates the transition processes.  Continued, but less frequent, case management by CMHS.  Facilitated re-entry into PMHS when needed  Unlimited duration |
| Filia et al. (2012), Australia | To identify criteria used by CMHS staff when determining patient suitability and barriers to discharging patients taking clozapine to private psychiatrist or PCP shared-care setting, | Mixed Methods: Cross-sectional survey – a clozapine transition questionnaire was sent to CMHS staff. | Alfred Community Mental Health Service (CMHS) | CMHS staff (n = 34)  Private psychiatrists (n = 29)  PCPs (n = 17) | Barriers deemed most significant on the questionnaire were, in order,: 1. Patient having a low level of personal organizational skills; 2. Patient having ongoing case management needs; 3. Less patient accountability in the private/PCP setting; 4. Patient’s level of medical compliance.  Additional barriers identified by participants were: Time required for transition process (n=4), lack of information about transition process (n=2), lack of support from the PCP/private psychiatry sector (n=2), lack of support from CMHS (including review services or resuming care transitioned patients) (n=6); amount of paperwork required (n=2); need to establish therapeutic relationships (n=3); clozapine side-effects (n=1); blood test and prescription authorization to allow patients to go on holidays (n=1). | Most important factors for transitions were patients are: Compliant with medication; 2. Independently attend appointments and blood tests; 3. Can access a suitable pharmacy for their medication; 4. Patient is happy with private care 5. Is physically able to get to the private service.  Additional facilitators were: CMHS support being available if necessary (n=2); patients has no current substance abuse (n=1); patient is able to afford change in treatment (n=1). | Shared Care Model for Clozapine | See above for Filia et al 2012. |
| Hamilton-West et al. (2017), United Kingdom | To evaluate a pilot service called the Primary Care Mental Health Specialist (PCMHS), which was developed to facilitate discharge from secondary to primary care for patients with stable and long-term mental health needs | Mixed Methods: Formative service evaluation – Interviews were conducted with all PCMHS employed in the pilot service, as well as a sample of service users. A brief online questionnaire was provided to professionals working alongside the service. Time and activity recording sheets were used to complete an economic analysis. | Secondary mental health services | Patients (n = 12, 5 male, M = 50.8 years (range 23-64 years), with stable, long-term mental health conditions)  PCMHSs (n=13, community psychiatric nurses and occupational therapists)  PCPs (n = 16)  Managers/Operations (n = 11)  Primary Care Link Workers (n = 7)  Psychiatrists (n = 6)  Mental Health Nurses (n = 3)  Practice Managers (n = 2)  Social  Workers (n = 1)  Others (n=3) |  | Respondents indicated PCMHS prevented relapse and/or readmission to secondary care | Primary Care Mental Health Specialist (PCMHS) | Primary Care Mental Health Specialist (PCMHS): supports client and PCP and facilitates the transition processes.  Direct therapy work from PCMHS (eg. psychoeducation, occupational therapy)  Facilitated re-entry into secondary care service when needed.  Unlimited duration. |
| Haslam et al. (2006), Canada | To describe the Transition into Primary Care Psychiatry (TIPP) clinical model, developed based on the CLIPP model | Program description of the TIPP mocdel | TIPP was implemented in a variety of outpatient mental health services in London and Thunder Bay, Ontario |  |  |  | Transition into Primary Care Psychiatry (TIPP) | Consultations with psychiatrist every 3-6 months based on need.  TIPP nurse: supports client and PCP and facilitates the transition process.  Community mental health nurse support every 1-3 months  CMHS services may be facilitated if necessary (e.g. case management)  Care plan and relapse signature developed with client, CMHS, and support network  Case conference with care providers and client at PCP office to complete transfer of care.  Unlimited duration |
| Holmes et al. (2005), Australia | To assess the relationship between care outcomes in patients with psychosis and patient histories of living accommodations | Quantitative: Retrospective accommodation history – demographic information, diagnosis, and accommodation history were obtained from patients with psychosis accepted for case management over a 12-month period. Two years later, patients were followed up with to determine continuity of care and discharge outcome. | Public psychiatric service - Inner West Area Mental Health Service | Patients with psychosis (N =  143, 99 males (M age 35.4 years) and 44 females (M age 45.3 years) | Patients with a history of homelessness were more likely to experience a discontinuity of care at discharge and less likely to be discharged to primary care |  |  |  |
| Horner & Asher (2005), Australia | To describe a shared-care protocol used by their program (which includes a General Practitioner Clinical Liaison Officer; GPCLO) and measure performance indicators (PCP satisfaction with protocol and patient mental health indicators) | Quantitative: Program description and evaluation – patients from the shared care program were given outcome measures, GPs were provided with surveys | Ryde Community Mental Health Service | Patients (n = 56, with diagnoses of schizophrenia (n = 34), bipolar disorder (n = 4), schizoaffective disorder (n = 4) and other diagnoses (n = 7))  PCPs (n = 13) |  | 10 PCPs reported the GPCLO as useful  9 PCPs reported the multidisciplinary care planning meeting was helpful  12 PCPs reported the management plan was helpful | Shared care protocol (based on the CLIPP model and a model used by the Hornsby Ku-ring-gai Division of General Practice (Sydney, Australia)) | Consultations with psychiatrist - frequency is negotiated.  General Practitioner Clinical Liaison Officer (GPCLO): supports client and PCP and facilitates the transition processes.  Care plan and relapse signature developed by PCP, client, client support system, case manager, psychiatrist and GPCLO.  Facilitate re-entry into CMHS when needed.  Care-planning meeting at PCP office with all care providers and discussion of identified potential problems with transfer.  Unlimited duration. |
| Hurley et al. (2021), United Kingdom | To evaluate whether the Recovery and Enablement Track (RET), a service for facilitating discharge from specialist mental health services improved discharge outcomes. | Quantitative: Prospective service evaluation – a group of patients that entered the RET were followed up to 12 months’ post-discharge. Recovery, well-being, and distress were measured at assessment, review, and discharge. | East and West Enfield Community Mental Health Teams | Patients (n = 86, 50% male, Median age = 45 (IQR = 36-53),  65% had schizophrenia spectrum disorder and 27% had Bipolar affective disorder diagnoses) |  |  | Recovery and Enablement Track (RET) | Key worker: conducts recovery focused working sessions with CMHS clients.  Option to complete several recovery-focused groups.  Facilitated re-entry into CMHS when needed  Duration: 6-12 months prior to CMHS d/c  Client-set recovery goals form basis of program |
| Jespersen et al. (2009), Australia | To describe the authors’ experience of facilitated discharge from specialist mental health services to primary care and review research evidence from the Consultation and Liaison in Primary Care (CLIPP) model and the primary care liaison model (PCL). | Quantitative: Literature review (CLIPP study - retrospective file review, PCL study - survey) and authors’ reflections | Specialist mental health services | CLIPP: Patients (n = 42, 19 female, 11 had symptoms of psychosis)  PCL: Patients (n = 10) |  | CLIPP study: Patients discharged to PCPs had  higher level of function (using Global Assessment of Functioning), Limited past depot prescription, Fewer contacts with triage during CLIPP, Fewer CLIPP contacts with family, less high prevalence disorders, Fewer medical conditions, less psychosocial stress, and trends towards: Female sex, more employment, less chronicity of symptoms, less behavioural symptoms, less current and past involuntary treatment on a community treatment order. | Consultation and Liaison in Primary Care (CLIPP)  Primary Care Liaison (PCL) | **CLIPP**  Bimonthly half-day consultation  psychiatric reviews every 6-12 months.  CLIPP Nurse: facilitates initial transition. provides outreach to patients, where attendance has been flagged.  Care plan and relapse signature developed by CMHS.  Case conference with care providers and client at PCP office to complete transfer of care.  Unlimited duration.  PCP mental health review and support every 2–4 weeks.  Attendance monitoring.  **PCL**  Secondary consultation - Frequency/availability of specific support from psychiatry is unclear  Work with clients on developing social networks and understanding of illness - Unclear parties involved  Care plan and relapse signature developed by case manager with PCP and patient input.  Facilitated re-entry into CMHS when needed  Patient and case manager have three meetings to prior to d/c  Duration: 3 months |
| Lester et al. (2012), United Kingdom | To explore service users' perspectives of early intervention services and primary care before and after discharge. | Qualitative: Longitudinal case study – semi-structured interviews were conducted with individuals with psychosis at two time points (before and after discharge) | Five early intervention services for psychosis | Service users  -Time 1: n = 63, M age = 23 years (range 18–33 years  -Time 2: n = 21, 66% male | Unexpected or abrupt discharges  Poor communication between early intervention services and primary care services  Feelings of being passed on/over by early intervention services | Planned and expected discharges  Service users feeling prepared for transition  Recognition of service user’s self-management ability  Personalized transition processes  Flexible timing for transitions  Previous strong and trusting relationships with PCP.  Good communication between service user, follow-up care and the early intervention service  PCPs need to recognize need for additional services and how to access them quickly. |  |  |
| Meadows et al. (2007), Australia | To describe CLIPP and supporting evidence  *CLIPP model described in earlier articles in 1998, 2003 | Quantitative: Program description and outcome evaluation: 62 patients, the majority with schizophrenia, were transferred over a period of two years from specialized mental health services into CLIPP care. They were observed for two years after transferred and a variety of outcome measures were administered to them before the transfer, at 12 months, and at 24 months post-transfer. | Community mental health services | Patients (n = 62, with psychiatric disorders  (schizophrenia most common) |  |  | Consultation-Liaison in Primary-Care Psychiatry (CLIPP) | Bimonthly half-day consultation  psychiatric reviews every 6-12 months.  CLIPP Nurse: facilitates initial transition. provides outreach to patients, where attendance has been flagged.  Care plan and relapse signature developed by CMHS.  Case conference with care providers and client at PCP office to complete transfer of care.  Unlimited duration.  PCP mental health review and support every 2–4 weeks.  Attendance monitoring. |
| Ramanuj et al. (2015), United Kingdom | To assess acute mental health service use by people discharged to primary care with SMI | Quantitative: Retrospective cohort study of 98 patients in two years following discharged to primary care from mental health services | Community Mental Health Centre (CMHT) in Lambeth, South London | Patients discharged to primary care: n=98 (48% male; median age = 42.5 (IQR = 36.0–58.0); 60.2% had schizophrenia spectrum psychoses; 20.4% had bipolar affective disorder)  Patients transferred to a different CMHT: n=92 (59.8% Male; median age = 45.0 (37.0–53.0); 68.5% had schizophrenia spectrum psychoses; 17.4% had bipolar affective disorder) | Admission for acute mental health care within the two years prior to transfer/discharge was associated with increased odds of requiring acute care and increased duration of care after transfer.  Requiring acute mental health care in the 2 years prior to discharge increased odds of re-referral by nearly four times.  Discharge on oral medication increased odds of re-referral by 14.78 | Discharged group had spent less time with CMHT, had spent less time receiving acute mental health services, were more likely to not be taking medications, and less likely to be taking depot medications. Discharged group also had lower median Health of the Nation Outcome Scale (HoNOS) scores (i.e., better functioning). |  |  |
| Rodenburg et al. (2004), New Zealand | To describe and evaluate a service designed to enable PCPs to provide care for individuals with SMI that were previously cared for by CMHSs. | Mixed Methods: Service evaluation – mental health consumers were interviewed at regular intervals after they entered the program. Quantitative outcome measures were used at regular outcomes to measure their condition and overall function. A cost comparison was made between services offered before and after entry to the mental health progarm | Specialist community mental health service | Patients (N=163, 27% had a mood disorder with elements of psychosis, and 44% had schizophrenia -spectrum disorders) | Uncertainty about PCP’s mental health/illness knowledge  Concerns about PCP appointment time constraints  Fears about PCP physical environment (unfamiliarity, stress of noise/location)  Concern about loss of contact with a  psychiatrist  Concerns about getting sufficient support for “non-clinical issues” | Transparency of information related to discharge planning  Patient involvement and ownership of their care plans  Patient not feeling pressure to transfer  Convenient PCP location  Not having to pay for PCP appointments  Welcoming/supportive PCPs, nurses, and receptionists at PCP offices  Ability to see PCP for physical and mental health | Wellington Mental Health Liaison Service | Primary care liaison worker (PCLW): supports clients through transition and provides consultation to PCPs after transfer  Care plan/relapse signature developed by PCLW, patients, and PCPs.  Education for all PC staff and resource manual for PCPs  Meetings coordinated as needed  Unlimited duration  Provision of PCP consults for free to patients |
| Röhricht et al. (2017), United Kingdom | To evaluate clinical outcomes of the enhance primary care (EPC) service | Quantitative: Retrospective service evaluation – data from 2818 patients and staff survey results were analysed | Secondary care | Patients (n = 2330, M age = 45.7 (range = 18 - 65); 46% male;  37.2% were diagnosed with a psychotic disorder.) |  |  | Enhanced Primary Care (EPC) | Regular psychiatrist support in consultative role  Primary Care Liaison Nurses (PCLN): supports client and PCP and facilitates the transition process  Ongoing recovery oriented support from mental health nurses  Peer support workers assist during d/c from EPC  Recovery Care Plan  Facilitated re-entry into secondary care when needed  Primary care education on SMI and management.  Duration: 1 year  Nurse administration of depot injections |
| Stangroom et al. (2014), New Zealand | To assess primary care engagement following discharge from CMHS | Mixed Methods: Retrospective audit – primary care teams for 55 service users discharged from community mental health services were asked to provide information regarding engagement with GPs | Four Community Mental Health Centres (CMHCs) in Auckland District Health Board | PCPs (n = 50) of discharged patients (n = 50, 56% male, M=43.9 years, SD = 11.5, 20% had a mental health condition with psychosis) | - High service needs - Lack of engagement with PCP - Some PCP comments highlighted poor communication between PCPs and CMHCs, difficulty contacting the CMHC for advice, and inability to attend to the needs of discharged individuals due to time limitations | One PCP clinic identified good communication and support from CMHC |  |  |

Appendix C. Completed data extraction table.

**Appendix D**

| **Barrier** | **Sources (*N* = 20)** |
| --- | --- |
| **Patient-related** |  |
| Patient stability | 3 |
| Care needs | 5 |
| Socioeconomic status | 3 |
| Engagement with treatment | 4 |
| Readiness for discharge | 2 |
| **Primary care capacity-related** |  |
| Accessibility and care context related-factors | 4 |
| PCP ability to meet patient needs | 4 |
| **Transition Process/Support-related** |  |
| Quality of communication and support across care settings | 3 |
| Work and time required to facilitate discharge | 1 |
| **Facilitators** | **Sources (*N*=20)** |
| **Patient-related** |  |
| Stability | 6 |
| Patient strengths | 4 |
| Readiness for discharge | 5 |
| **Primary care capacity-related** |  |
| Accessibility and care context-related factors | 3 |
| PCP ability to meet mental health care needs | 4 |
| **Transition Process/Support-related** |  |
| Discharge planning process | 4 |
| Communication and support across services | 7 |

Appendix D: Barriers and Facilitators of Discharge

**Appendix E**

| **Program** | **Authors cited ; Country** | **Consultations with psychiatrists** | **Novel role supporting transition process** | **Additional mental health supports** | **Care plans with relapse signatures and Interventions** | **Facilitated re-entry into CMHS** | **Formal training provided** | **Meetings to prepare for d/c** | **Other** |
| --- | --- | --- | --- | --- | --- | --- | --- | --- | --- |
| Transition into Primary Care Psychiatry (TIPP) clinical model. | Haslam et al., (2006), Canada | Every 3-6 months based on need. | TIPP nurse: supports client and PCP and facilitates the transition process. | Community mental health nurse support every 1-3 months  CMHS services may be facilitated if necessary (e.g. case management) | Developed with client, CMHS, and support network | - | - | Case conference with care providers and client at PCP office to complete transfer of care | - |
| The Consultation and Liaison in Primary Care Psychiatry (CLIPP) | Meadows et al., (2007), Jespersen et al., (2009); Australia | Bimonthly half-day consultation  psychiatric reviews every 6-12 months. | CLIPP Nurse: facilitates initial transition. provides outreach to patients, where attendance has been flagged. |  | Developed by CMHS | - | - | Case conference with care providers and client at PCP office to complete transfer of care | PCP mental health review and support every 2–4 weeks.  Attendance monitoring |
| *Recovery and Enablement Track | Hurley et al., (2021); United Kingdom | NA | Key worker: conducts recovery focused working sessions with CMHS clients. | Option to complete several recovery-focused groups. | NA | yes | NA | NA | Client-set recovery goals form basis of program |
| Enhanced Primary Care (EPC) Pathway | Rohricht et al., (2017);  United Kingdom | Regular psychiatrist support in consultative role | Primary Care Liaison Nurses (PCLN): supports client and PCP and facilitates the transition process. | Ongoing recovery oriented support from mental health nurses  Peer support workers assist during d/c from EPC | Recovery Care Plan | yes | Primary care education on SMI and management. | – | Nurse administration of depot injections |
| Primary Care Liaison | Jespersen et al., (2009);  Australia | Secondary consultation** | - | work with clients on developing social networks and understanding of illness.*** | Developed by case manager with PCP and patient input. | yes | - | Patient and case manager have three meetings to prior to d/c | . |
| Wellington Mental Health Liaison Service | Rodenberg et al, (2004); New Zealand | - | Primary care liaison worker (PCLW): supports clients through transition and provides consultation to PCPs after transfer | - | Developed by PCLW, patients, and PCPs. | - | Education for all PC staff and resource manual for PCPs | Meetings coordinated as needed | Provision of PCP consults for free to patients  . |
| Modified shared care protocol | Horner et al., (2005); Australia | Yes - frequency is negotiated | General Practitioner Clinical Liaison Officer (GPCLO): supports client and PCP and facilitates the transition processes. | - | Developed by PCP, client, client support system, case manager, psychiatrist and GPCLO | yes | - | Care-planning meeting at PCP office with all care providers and discussion of identified potential problems with transfer. |  |
| Primary Care Mental Health Specialist (PMHS)  Service | Hamilton-West et al., (2017);  United Kingdom |  | Primary Care Mental Health Specialist (PCMHS): supports client and PCP and facilitates the transition processes. | Direct therapy work from PCMHS (eg. psychoeducation, occupational therapy) | NA | yes | - | - | - |
| Shared Care Model for Clozapine | Filia et al., (2012), Filia et al, (2013); Australia | Completes a review every 6 months or when required | clozapine coordinator: supports client and PCP and facilitates the transition processes. | Continued, but less frequent, case management by CMHS. | - | yes | - | - |  |
| Planned Discharge Process | Backus et al. (2008), Australia |  |  | Connections made to community support services and recreation prior to d/c | Developed by care team at CMHS**** |  | - | Meeting of CMHS staff to determine potential for client d/c | Ensured plan for PCP follow-up |
| PARTNERS (develoPing integrAted primaRy  care for paTieNts with sERiouS mental illness) | Baker et al., (2019), United Kingdom | Psychiatrist consultation available if needed | Care Partner:  liaises between levels of care and provides direct support to clients  Supervisor: conducts meetings with care partners bimonthly to consult on care. | 20-90 minute intervention sessions involving coaching, goal setting, and motivational interviewing and psychoeducation. | Yes. |  | Formal training provided to care partner and supervisor |  | Shared records |

*Program completed by clients while still under the care of CMHS

** Frequency/availability of specific support from psychiatry is unclear

*** Unclear parties involved

**** Unclear whether care plan included detailed explanation of relapse signature

Appendix E. Coding table for research question #2.
